# Synchronous Chronic Lymphocytic Leukemia/Small Lymphocytic Lymphoma and Metastatic Squamous Cell Carcinoma of the Cervix Involving the Coronary Arteries Leading to Acute Myocardial Infarction

**DOI:** 10.1155/2020/6192754

**Published:** 2020-02-21

**Authors:** Michelle Bernshteyn, Alexandria Smith-Hannah, Amit S. Dhamoon

**Affiliations:** SUNY Upstate Medical University Hospital, 750 E Adams St., Syracuse, NY 13210, USA

## Abstract

A 66-year-old woman presented to the hospital with a one-month history of shortness of breath, fatigue, and postmenopausal vaginal bleeding and a one-week history of chest pain. This case report discusses the rare synchronous occurrence of two different malignancies in the setting of non-ST segment elevation myocardial infarction and the relation between these unfortunate events. Besides the case presented in this report, there have been only 13 reported cases of synchronous chronic lymphocytic leukemia/small lymphocytic lymphoma (CLL/SLL) associated with metastatic squamous cell carcinoma. While it is well known that malignancy causes a hypercoagulable state, there are other mechanisms which may have contributed to the patient's myocardial ischemia including external vascular compression, tumor lysis syndrome, and anemia. This case report discusses the rarity of synchronous malignancies but the importance of understanding and consideration of cardiac events in this population.

## 1. Introduction

Synchronous tumors are rare. Patients who present with CLL/SLL associated with an aggressive metastatic SCC are more vulnerable to malignant spread, recurrence, and death as compared to individuals without an associated secondary malignancy [1]. It is known that cancer cells impact the coagulation cascade leading to a hypercoagulable state, thrombus formation and propagation, and anemia which can lead to venous thromboembolism. The risk of arterial ischemic events due to underlying malignancy is not well described in the literature. According to a study involving a multicenter prospective registry of patients with venous thromboembolism, arterial ischemic events appeared earlier as compared to the general population and contributed to death. Venous thromboembolic events are more common in patients with malignancy. The types of arterial ischemic events were not specified [2]. In another study, it was demonstrated that the rate of cerebral ischemic events in patients with malignancy was not statistically significant as compared to patients without malignancy [3]. The relationship of malignancy resulting in myocardial ischemia is not well studied. In a reported case, the autopsy of a patient diagnosed with CLL demonstrated infiltration of malignant cells into coronary arteries extending to their adventitial and medial layers ultimately contributing to acute myocardial infarction [4].

## 2. Case Presentation

The patient discussed in this case is a 66-year-old female with an unknown past medical history since the patient had not been seen by a physician for approximately 43 years. The patient was transferred to our university hospital from a community hospital for management of chest pressure, shortness of breath, and vaginal bleeding. The patient presented with a one-week history of chest pressure. This was described as a feeling of tightness which occurred several times over the span of one week and was not associated with exertion. Her shortness of breath had been progressively worsening over one month. Prior, to one month ago, she reportedly was able to ambulate approximately 100 yards without difficulty. By the time of her presentation, the patient stated that she was only able to ambulate approximately 20 yards due to shortness of breath on exertion. The patient stated that her vaginal bleeding was similar in volume as compared to previous menstrual cycles and had been occurring approximately two times per week. Her past surgical history was significant for hiatal hernia repair and tonsillectomy, both during adolescence. Furthermore, she smoked once pack of cigarettes daily for fifteen years and has quit two months prior to admission. She had two aunts who passed away due to myocardial infarctions at unspecified ages. On review of systems, the patient reported decreased appetite, fatigue, diaphoresis, cough, shortness of breath, chest pressure, nausea, vomiting, dysuria, hematuria, vaginal bleeding, pallor, light-headedness, and a 20-pound weight loss over several months. Physical exam at time of admission was unremarkable.

In the Emergency Department, the patient underwent an EKG which demonstrated Q waves in inferior leads. Her troponin T was 0.61 ng/mL. Subsequent troponin T levels were measured, and within 24 hours, this increased to 5.88 ng/mL. Therefore, the patient was diagnosed with a non-ST elevation myocardial infarction and cardiology was consulted.

The patient was found to have a hemoglobin level of 4.5 g/dL and hematocrit level of 18.3%. It was presumed that this anemia was secondary to the patient's reported vaginal bleeding. Anticoagulation for NSTEMI was therefore not recommended. On further evaluation, the patient was found to have an elevated white blood cell count of 39,900 cells/mm^3^, elevated uric acid of 8.2 mg/dL, hyperkalemia as high as 5.6 mmol/L, hyperphosphatemia as high as 4.7 mg/dL, and an elevated creatinine of 2 mg/dL. Thus, she was suspected to have tumor lysis syndrome given these multiple electrolyte aberrancies. The patient was transfused with two units of packed red blood cells and rasburicase was administered.

A transvaginal ultrasound was performed which demonstrated a prominent cervical mass compressing the left ureter and associated hydronephrosis. Given the acute kidney injury as diagnosed due to a rising creatinine to 2 mg/dL, nephrostomy tube insertion was initially recommended but not placed due to the patient's poor prognostic factors. She was neither anuric nor oliguric. The patient underwent cervical biopsy and was found to have invasive squamous cell carcinoma of the cervix. The cervical cancer had eroded a 1.5 cm hole into the bladder extending down the anterior vaginal wall approximately at the introitus, according to the physical examination performed by the Gynecology team.

Due to NSTEMI, transthoracic echocardiogram was performed demonstrating an ejection fraction of 30-35%, increased PA systolic pressure, grade III diastolic dysfunction, and mitral and tricuspid regurgitation. Cardiac catheterization was performed one day after admission demonstrating a total occlusion of left anterior descending and distal left circumflex arteries. Lack of collateral vessel perfusion suggests that occlusion was not attributed to chronic atherosclerotic changes and more likely due to acute blockage. In addition to initiating a continuous heparin infusion at this time, the patient was offered revascularization but declined. Risk of sudden death was thoroughly explained but the patient was not experiencing chest pain and wanted to focus on managing her vaginal bleeding rather than revascularization.

The patient proceeded to have a sustained elevated heart rate but decreased blood pressure likely due to cardiogenic shock and atrial fibrillation with rapid ventricular response. Heparin was discontinued, and digoxin was initiated.

On day seven, she demonstrated ventricular tachycardia with ambulation, losing consciousness and subsequently going into asystole. ACLS was initiated, the patient was defibrillated and resuscitated, and the patient was intubated and supported on vasopressors. A family meeting was held, and it was decided that comfort care measures would be pursued. The patient died five minutes after extubation.

**Figure 1:**
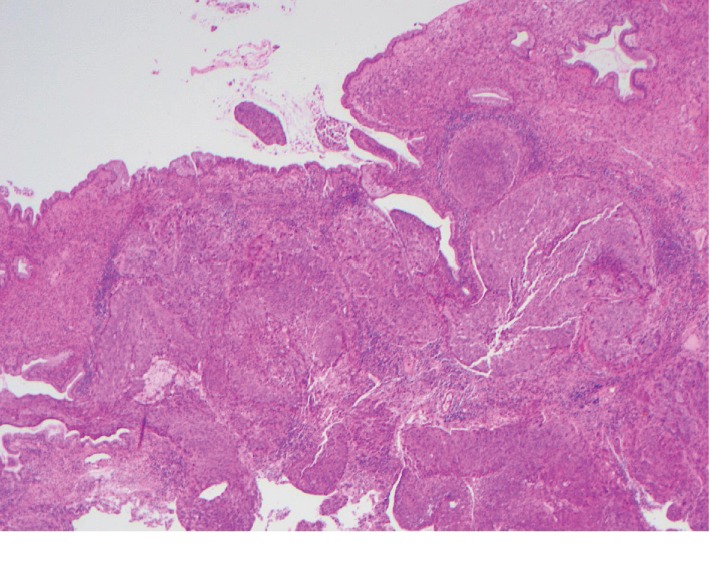
Invasive squamous cell carcinoma with benign endocervical gland present in the upper right hand corner. This was associated with extensive necrosis.

After the patient's demise, pathological results were finalized. Based on the surgical pathology report of the cervical mass, moderately differentiated invasive squamous carcinoma with extensive necrosis was seen (Figure 1).

**Figure 2:**
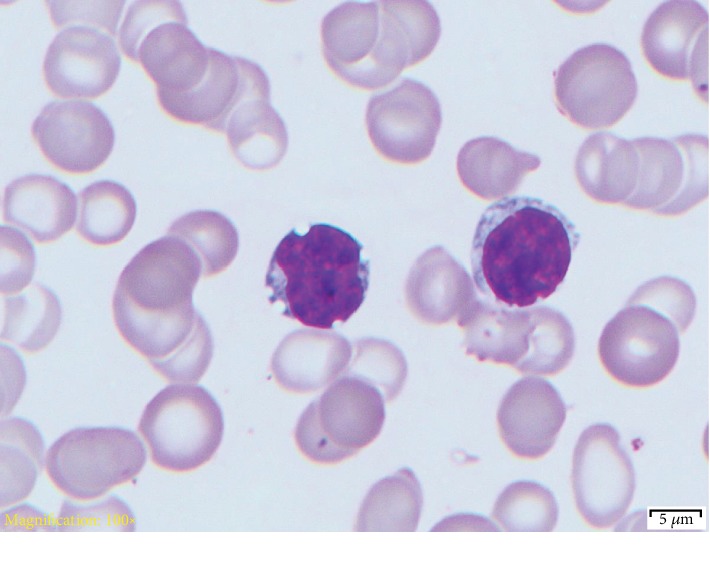
Small- to medium-sized lymphocytes with round to slightly indented nuclear contours. There was inconspicuous nucleoli and minimal amount of cytoplasm.

Hemopathology report described that flow cytometry analysis demonstrated a clonal B cell population most consistent with CLL/SLL. Peripheral smear confirmed this diagnosis (Figure 2). FISH study demonstrated deletion of 11q and interstitial deletion of 13q (67-73% of cells). Deletions of 11q, as seen in this case, are observed in approximately 8-19% of CLL cases [5]. Furthermore, deletions of 13q, again seen in this case, are demonstrated in approximately 68% of CLL cases [5]. Deletions are a poor prognostic marker thus suggesting evolution of the cell line in this patient. Monosomies 11, 13, and 14 were present in 3-7.5% of the cells, but there was no evidence of trisomy 12, deletion of 17p, or t (11; 14).

## 3. Discussion

There are approximately 13 previously reported cases of synchronous CLL/SLL and invasive squamous cell carcinoma. On thorough review, all these cases involved male patients who ranged from 44 to 83 years of age [1]. Another case report, written seven years prior, stated that there have only been 11 reported cases of cutaneous SCC metastatic to tissues involving CLL/SLL. It appears as though certain malignancies, such as renal cell carcinoma and lymphoma, are more susceptible to a second malignancy [6].

Furthermore, all involved squamous cell carcinoma of the skin. Areas involved included the nose, ears, face, scalp, and/or neck [7]. This is the first case which involves a female patient and a squamous cell carcinoma of a site other than the skin.

It has been suggested that the mechanism underlying the development of synchronous malignancies may be the result of cancer-to-cancer metastasis which is influenced by nonmodifiable factors such as age and genetic susceptibility or modifiable factors such as exposure to carcinogenic agents via environment or lifestyle habits. Furthermore, cancer-to-cancer metastasis can be investigated by using specific immunohistochemical profiles [1]. Although many theories have been postulated, the exact mechanism has yet to be established.

In this case presentation, the patient reportedly smoked a pack of cigarettes daily for 15 years prior to admission. Furthermore, the patient had two aunts who suffered myocardial infarctions at unspecified ages. It is important to be aware of these risk factors to help diagnose and guide treatment. What complicated this case further is that the patient had not been seen by a health care provider for forty-three years. It is possible that the outcome could have been prevented with routine screening tests.

In this case, there are various possible mechanisms in which a patient with synchronous malignancies could have suffered a myocardial infarction. This includes the hypercoagulable state associated with the presence of malignancy, anemia contributing to demand ischemia, infiltration of coronary arteries by cancerous cells, and the impact of tumor lysis syndrome specifically causing neuromuscular irritability and elevated potassium leading to cardiac dysrhythmia.

Malignancy is an independent risk factor for thrombosis. It has been suggested that there is an upregulation of inflammatory cytokines and hemodynamic changes from increased nitric oxide which contribute to the hypercoagulable state [8]. Furthermore, tumor cells activate fibrinolytic pathways and platelets [9]. Even though not all of the mechanisms are completely understood, it is evident that malignancy can lead to clot formation. It has been suggested that endocrine/metabolic changes (such as anemia), hemodynamic changes, mental stress, and cancer therapeutics themselves contribute to vasospasm, erosion, plaque hemorrhage, and embolus [10].

Coincidentally, thromboembolism is often one of the earliest signs that a malignancy is present, as suggested by this case report [9]. Presumably, the presence of two cancers further increases this risk. Tumor cells activate the coagulation system due to the production of inflammatory cytokines, acute phase reactants, and procoagulant substances [9].

The patient presented in this case report presented with a one-month history of postmenopausal vaginal bleeding. This was the presumed cause of her profound anemia. At transfer, the patient demonstrated a hemoglobin level of 4.5 g/dL and a hematocrit level of 18.3%. Red blood cells have the responsibility of oxygen delivery to muscles such as a myocardium. It has been shown that anemia is a known risk factor for ischemic heart disease and that the mortality rate is higher in patients with anemia in the setting of ischemic heart disease as compared to the latter alone [11]. Thus, the anemia may not have solely caused the patient's death, but it perhaps contributed to it.

A previous case report highlights an interesting correlation between CLL and acute myocardial infarction. The mechanism for which chronic lymphocytic leukemia leads to impaired flow through the coronary arteries is proposed as different compared to that of an atherosclerotic plaque or thrombus. Tumor encases the coronary arteries causing external compression of the vessel, whereas atherosclerotic plaque or thrombus precipitates occlusion within the vessel [1]. This was demonstrated on autopsy in which malignant cells extended into the tunica adventitia and tunica media. Even though this complication has been previously documented, it is extremely rare. The patient in this case report underwent cardiac catherization which demonstrated occlusion of the left anterior descending and distal left circumflex arteries. However, there is no way to know if CLL cells infiltrated the arteries externally because no autopsy was performed. Given the patient's prominent but nonextensive smoking history, this may be a likely contributing factor.

Tumor lysis syndrome occurs when there is mass destruction of tumor cells leading to electrolyte abnormalities. It most often occurs because of initiating of chemotherapeutic agents. However, tumor lysis syndrome can occur spontaneously [12]. This is a rare occurrence but is another feature which makes this case report unique. The patient was presenting to the hospital with unknown underlying malignancies and was not actively receiving chemotherapeutic therapy. However, there was evidence to support tumor lysis syndrome with hyperphosphatemia, hyperkalemia, and resultant hypocalcemia. This quick electrolyte shift leads to neuromuscular irritability and tetany [13]. Furthermore, cellular mass destruction causes potassium to leak out of the intracellular space into the extracellular space, affecting the cellular membrane of cardiac tissue. This cascade of events can ultimately lead to ventricular arrhythmias [13]. On presentation, the patient's potassium level was elevated at 5.5 mmol/L. The instability caused by tumor lysis syndrome contributed to the cardiac malfunctions that the patient experienced. Even though rasburicase has been demonstrated to be effective in pediatric and adult populations for management of uric acid levels in tumor lysis syndrome, it is interestingly only approved by the Federal Drug Administration for use in the pediatric population [14].

Several key points can be learned from this rare case presentation. First, healthcare providers should encourage patients to follow up with recommended screenings. This is extremely important to prevent outcomes such as those seen in this patient [15]. Second, it is important to realize that albeit rare, synchronous malignancies can occur. In situations such as this, healthcare providers should aim to detect signs of tumor lysis syndrome. This can help prevent the onset of cardiac arrhythmias. Third, it is important to note that malignancy is a prominent risk factor for thromboembolic events and concurrent anemia can promote ischemic heart disease. More specifically in this patient, CLL has been demonstrated to cause external compression and infiltration of coronary arteries. This is a unique case presentation which can help guide treatment to shorten the length of hospitalization and ultimately promote longevity.
